# Effects of Long-Term Oral Administration of Arachidonic Acid and Docosahexaenoic Acid on the Immune Functions of Young Rats

**DOI:** 10.3390/nu5061949

**Published:** 2013-05-29

**Authors:** Sachiko Juman, Michio Hashimoto, Masanori Katakura, Takayuki Inoue, Yoko Tanabe, Makoto Arita, Tomohiro Miki, Osamu Shido

**Affiliations:** 1 School of Pharmacy and Pharmaceutical Sciences, Mukogawa Women’s University, 11-68 Koshien Kyuban-cho, Nishinomiya 663-8179, Japan; E-Mails: manju129@mukogawa-u.ac.jp (S.J.) tomomiki@mukogawa-u.ac.jp (T.M.); 2 Department of Environmental Physiology, Shimane University Faculty of Medicine, 89-1 Enya-cho, Izumo 693-8501, Japan; E-Mails: katakura@med.shimane-u.ac.jp (M.K.); tinoue@med.shimane-u.ac.jp (T.I.); tanabey@med.shimane-u.ac.jp (Y.T.); o-shido@med.shimane-u.ac.jp (O.S.); 3 Department of Health Chemistry, Graduate School of Pharmaceutical Sciences, University of Tokyo, 7-3-1 Hongo, Bunkyo-ku, Tokyo 113-0033, Japan; E-Mail: marita@mol.f.u-tokyo.ac.jp

**Keywords:** natural killer cell, polyunsaturated fatty acid, prostaglandin E_2_, prostaglandin D_2_, eicosanoids

## Abstract

Natural killer (NK) cells have many functional activities, including cytotoxicity and the capacity to produce cytokines and chemokines. NK cell activity is regulated partly by eicosanoids, which are produced from arachidonic acid (ARA) and eicosapentaenoic (EPA) acid. In this study, we investigated the effects of long-term therapy with ARA or docosahexaenoic acid (DHA) on the cytotoxic effects of the NK cells of young rats, which were fed on a nonfish oil diet for two generations. Control oil, ARA (240 mg/kg BW/day) or DHA (240 mg/kg BW/day) were orally administrated to the rats for 13 weeks before determining the cytotoxic activity of NK cells from the spleen against YAC-1 mouse lymphoma cell line, as well as the plasma levels of docosanoids or eicosanoids and inflammatory cytokines. Long-term ARA administration significantly suppressed the cytotoxic activity of NK cells. Moreover, ARA administration significantly increased the plasma levels of ARA, prostaglandin (PG) E_2_, and PGD_2_. However, DHA administration did not produce any different effects compared with those in the control rats. Furthermore, the inflammatory cytokine levels were not affected by the administration of ARA or DHA. These results suggest that long-term ARA administration has an inhibitory effect on the tumor cytotoxicity of NK cells in rat spleen lymphocytes owing to the enhanced synthesis of PGE_2_ and PGD_2_ from ARA because of the elevated plasma ARA levels in young rats.

## 1. Introduction

Natural killer (NK) cells are a type of lymphocyte that participates in innate immunity. The function of NK cells in innate immunity is crucial for combating viral infection and for destroying cancer cells [1,2]. Recent studies have demonstrated that NK cells have a direct cytotoxic activity against cancer cells, and they can regulate the adaptive immune response via cytokine production [3,4,5]. Thus, NK cells have important roles during the initial responses to viruses and cancer cells. A low NK cell activity is associated with an increased risk of carcinogenesis [6]. Therefore, the focus of recent cancer therapies has been in enhancing the NK cell activity through the development of chemical compounds. However, inflammation is a critical component of tumor progression and tumor microenvironments are controlled by inflammatory cells. Tumor cells activate specific signaling molecules in the innate immune system, including cytokines, chemokines, and their receptors during invasion, migration, and metastasis [7,8,9]. Therefore, recent cancer therapies have targeted immune modulation [10,11].

Polyunsaturated fatty acids (PUFAs) such as arachidonic acid (ARA), docosahexaenoic acid (DHA), and eicosapentaenoic acid (EPA), are involved in the inflammatory process. Inflammatory cytokines and tumor cells induce PUFA metabolizing enzymes such as secreted phospholipase A_2_ (sPLA_2_) and cyclooxygenase (COX)-2 [12]. ARA, a long-chain omega-6 fatty acid, is stored in the cell membrane phospholipids. ARA is released from the membrane phospholipids by sPLA_2_ and cytoplasmic phospholipase A_2_ (cPLA_2_), and this released free ARA can form eicosanoids via COX-1 and -2, lipoxygenases (LOXs), or cytochrome P450 monooxygenases [13,14]. ARA-derived eicosanoids such as prostaglandins (PGE_2_, PGF_2α_, PGD_2_), prostacyclin (PGI_2_), or thromboxane A_2_ and hydroxyeicosatetraenoic acid (HETE) have well-recognized roles in inflammation [15]. In fact, prolonged administration of non-steroidal anti-inflammatory drugs that can inhibit COX are associated with a reduced risk of developing several types of cancers [16]. In contrast, long-term ARA administration is associated with the development of cancer or the stimulation of inflammatory conditions [17,18,19].

DHA and EPA, which are omega-3 fatty acids, have a variety of anti-inflammatory and immune-modulating effects. In addition, these fatty acids are oxidized by COX, LOXs, or cytochrome P450 monooxygenases to produce docosanoids and EPA-derived eicosanoids, which have anti-inflammatory effects [20]. Various biological effects of omega-3 fatty acids have been demonstrated in several feeding studies with humans and animals using fish or fish oil supplements. In humans, omega-3 fatty acids inhibit the production of cytokines involved with chronic inflammatory and autoimmune diseases [21]. Dietary omega-3 fatty acids are considered to prevent inflammation through a variety of activities linked to the inhibition of ARA-derived eicosanoid-mediated effects, anti-inflammatory properties, and the inhibition of cytokines and ARA-derived eicosanoid synthetic enzymes by competitive inhibition. The typical ARA-derived eicosanoid, PGE_2_ is a potent regulator of inflammation as well as innate and adaptive immune responses, which contributes to immune evasion during malignancy. It is known that PGE_2_ downregulates interleukin (IL)-2-activated lymphokine-activated killer (LAK) cell cytotoxicity through EP2 receptors [22,23]. Moreover, PGE_2_ substantially inhibits IFN-γ production as well; it reduces the amount of IFN-γ and TNF-α secretion by NK cells. Similarly, IFN-γ secretion is suppressed by PGE_2_ in human NK cells in a dose-dependent manner [24].

The aim of the present study was to determine the relationships among the plasma levels of eicosanoid or docosanoid, inflammatory cytokines, and NK cell activity in long-term ARA- and DHA-administered young rats. In this study, we measured the plasma levels of eicosanoids and docosanoids using liquid chromatography tandem mass spectrometry (LC-ESI-MS/MS) because long-term administration of ARA and DHA might affect the levels of eicosanoids and docosanoids. Changes in the levels of eicosanoids and docosanoids modulate the inflammatory response, as described above; thus, we also investigated whether NK cell activity was affected by long-term oral administration of ARA and DHA. We used YAC-1 as a target cancer cell to evaluate NK cell activity. YAC-1 is a murine T-lymphoma cell line, which was established from a tumor induced by Moloney sarcoma virus in A/Sn mice. YAC-1 cells do not express major histocompatibility complex (MHC) class I molecules, and they are efficiently killed by NK cells. Moreover, YAC-1 has been used to study the cytotoxic activities of NK cells and LAKcells, which are induced by IL-2 from NK cells, along with factors that affect the cytotoxic activities of these and other lymphocytes [25,26].

## 2. Materials and Methods

### 2.1. Animals Used in the Experiments

All animal experiments were performed in accordance with the procedures outlined in the Guidelines for Animal Experimentation of Shimane University compiled from the Guidelines for Animal Experimentation of the Japanese Association for Laboratory Animal Science.

The animal room was maintained at 23 °C with a relative humidity 50%–55%. The light/dark cycle was maintained at 12 h intervals. Young Wistar rats (5 weeks old) were used in the study, which were the offspring of rats maintained for two generations on a nonfish oil diet (Funabashi Farm, Japan). The mean initial weight of rats was 214.6 ± 18.5 g. These rats were divided into three groups: control group, ARA group (the triglyceride form of ARA rich oil: 240 mg/kg BW/day), and DHA group (the triglyceride form of DHA rich oil: 240 mg/kg BW/day). Control rats were administrated a basic mixture of oil (beef fat:soybean oil:rape seed oil = 2:1:1). Each oil mixture was orally administered to the rats (*n* = 8 in each group) for 13 weeks. The fatty acid compositions of the oils administered are summarized in Table 1.

**Table 1:** Composition of fatty acids in control, ARA, DHA oil.

(mol%)	Control	ARA	DHA
PLA	13.8 ± 0.01	6.95 ± 0.00	29.8 ± 0.03
STA	13.8 ± 0.01	5.91 ± 0.00	8.10 ± 0.04
OLA	42.5 ± 0.03	5.31 ± 0.00	16.3 ± 0.01
LA	20.0 ± 0.02	9.38 ± 0.01	1.96 ± 0.01
ARA	ND	45.1 ± 0.04	2.49 ± 0.02
EPA	0.13 ± 0.01	0.52 ± 0.00	6.61 ± 0.00
DPA *n*-3	ND	ND	1.17 ± 0.01
DHA	ND	ND	32.6 ± 0.03

Note: PLA, palmitic acid; STA, stearic acid, OLA, oleic acid; LA, linolenic acid; ARA, arachidonic acid; EPA, eicosapentaenoic acid; DPA, docosapentaenoic acid; DHA, docosahexaenoic acid; ND, not detected.

### 2.2. Cell Culture

The YAC-1 mouse lymphoma cell line was provided by Riken Bio Resource Center through the National Bio-Resource Project of MEXT (Japan). Cells were cultured at 37 °C and 5% CO_2_ in RPMI-1640 medium with 10% FBS, 100 U/mL penicillin, and 100 µg/mL streptomycin. The cells were maintained in continuous suspension and cultured in the complete culture medium. YAC-1 cells were used to test the NK cytotoxic activity because of their notable sensitivity to NK cells.

### 2.3. Isolation of Rat Spleen Lymphocytes

The spleens were collected from rats who were administered the control, ARA, or DHA oils. These spleen samples were mashed through a strainer and the erythrocytes were removed using Lympholyte-Rat (Cedarlane, Burlington, Canada). Spleen lymphocytes were washed twice with Hank’s balanced salt solution and suspended in RPMI-1640 medium with 1% FBS. The number of lymphocytes in each sample was determined using a blood-cell counting chamber. The viability of lymphocytes was determined by trypan blue exclusion.

### 2.4. Cytotoxic Activity of NK Cell Assay

The NK cell activity was detected using freshly isolated spleen lymphocytes. The target cells used to determine the NK cell cytotoxicity were YAC-1 cells. Mixtures of spleen lymphocytes (1.5 × 10^5^, 7.5 × 10^4^, 3.75 × 10^4^/well/100 μL) and YAC-1 cells at 3 × 10^4^ cells/well/100 μL (lymphocytes:YAC-1 = 5:1, 2.5:1, 1.25:1) in RPMI-1640 with 1% FBS were incubated at 37 °C in a 5% CO_2_ incubator for 24 h. Subsequently, the cytotoxic activity levels of the NK cells in spleen lymphocytes were measured using a lactate dehydrogenase (LDH) Cytotoxicity Detection Kit (TaKaRa, Otsu, Japan). The cytotoxicity was measured on the basis of the amount of LDH released by damaged cells into the cell culture supernatant. LDH present in the culture supernatant participates in a coupled reaction that converts yellow tetrazolium salt into a red formazan product. The enzyme activity levels, which were measured as the absorbance at 490/620 nm using a microplate reader, were correlated with the number of damaged cells in the culture. The maximum release was the lysis achieved after adding Triton X-100 (final concentration 1%). The spontaneous release of LDH was determined by incubating labeled target cells in the absence of effector cells.

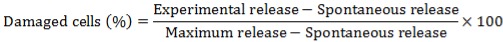



### 2.5. Sample Preparation for the Analysis of Fatty Acid Metabolites

Plasma samples were adjusted to 67% methanol and stored at −30 °C. The samples were centrifuged at 5,000 × *g* for 10 min at 4 °C to remove precipitated proteins. Thereafter, the supernatants were diluted with ice-cold distilled water and adjusted to 10% (v/v) methanol. Internal standards (5 ng of PGE_2_-*d*_4_, PGD_2_-*d*_4_, 5-HETE-*d*_8_, and AA-*d*_8_) were added to each sample. Samples were acidified to pH 4.0 using 0.1 M HCl and placed immediately in preconditioned solid phase extraction cartridges (Sep-Pak C18, Waters) to extract the fatty acid metabolites. Sep-Pak cartridges were washed with 20 mL MilliQ water and 20 mL *n*-hexane, and subsequently, the finally fatty acid metabolites were eluted with 10 mL methyl formate.

### 2.6. LC-ESI-MS/MS-Based Analysis

The fatty acid metabolites in the plasma were measured as described previously with a slight modification [27]. HPLC was combined with ESI-MS in a TSQ Quantum mass spectrometer (Thermo Fisher Scientific K.K., Japan). HPLC was conducted at 30 °C using a Luna 5u C18 (2) 100 Å LC column (150 × 2.0 mm, Phenomenex, USA). Samples were eluted with a mobile phase that comprised acetonitrile:methanol (4:1, v/v) and water:acetic acid (100:0.1, v/v) in a 27:73 ratio for 5 min, which was ramped up to a 100:0 ratio after 25 min, and subsequently held for 10 min at a flow rate of 0.1 mL/min. MS/MS analyses were conducted in the negative ion mode, and the fatty acid metabolites were detected and quantified by selected reaction monitoring (SRM). The conditions used for the detection of each compound by SRM are listed in Table 2. The peaks were selected and their areas were calculated using the Xcalibur 2.1 (Thermo Fisher Scientific K.K.).

**Table 2:** Selected reaction monitoring (SRM) transitions of fatty acid metabolites.

Compound	SRM Transition (*m/z*)	Compound	SRM Transition (*m/z*)	Compound	SRM Transition (*m/z*)
ARA	303 > 259	EPA	301 > 257	DHA	327 > 283
PGE_2_	351 > 271	5-HEPE	317 > 115	7-HDoHE	343 > 141
PGD_2_	351 > 271	12-HEPE	317 > 179	10-HDoHE	343 > 153
PGF_2α_	353 > 193	15-HEPE	317 > 219	14-HDoHE	343 > 193
5-HETE	319 > 115	18-HEPE	317 > 259	17-HDoHE	343 > 245
12-HETE	319 > 179	RvE2	333 > 115	PD1	359 > 153
15-HETE	319 > 219	AA-*d*_8_	311 > 267	PGE_2_-*d*_4_	355 > 275
PGD_2_-*d*_4_	355 > 275	PGF_2α_-*d*_4_	357 > 197	5-HETE-*d*_8_	327 > 116

Note: PG, prostaglandin; HETE, hydroxyeicosatetraenoic acid; HEPE, hydroxyeicosapentaenoic acids; RvE2, ResolvinE2; HDoHE, hydroxydocosahexaenoic acid; PD1, Protectin D1.

### 2.7. Determination of Cytokine Levels

The plasma concentrations of IL-1β, IL-4, IL-6, IL10, IL-13 and tumor necrosis factor (TNF)-α were measured using the Bio-Plex system, which combines a sandwich immunoassay with Luminex ﬂuorescent bead-based technology (Bio-Rad Laboratories, Inc. Hercules, CA, USA).

### 2.8. Analysis

The results obtained using at least 6–8 animals were expressed as the mean ± standard error (S.E.). Statistical differences in the NK cell activity were determined using a one-way ANOVA with Dunnett’s *post hoc* test, whereas the statistical differences in fatty acid metabolites were tested using a Bonferroni test. Differences were considered statistically significant at *p* < 0.05.

## 3. Results

### 3.1. Measurement of Cytotoxic Activity of NK Cells

**Figure 1:**
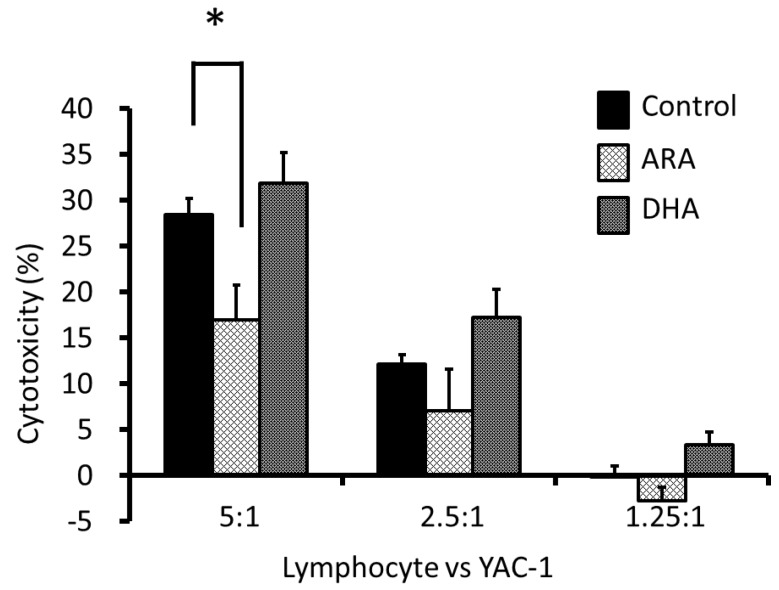
Cytotoxicity of natural killer (NK) cells in rat spleen lymphocytes against YAC-1 cells. The cytotoxicity levels in YAC-1 cells caused by NK cells in spleen lymphocytes after 24 h coculture in the *y*-axis. The ratio of the mixture of lymphocytes and YAC-1 is presented on the *x*-axis. The relative cytotoxicity levels are expressed as the mean ± S.E. (*n* = 6). * significant at *p* < 0.05 *vs.* rat lymphocytes in the control group using a one-way ANOVA followed by Dunnett’s test.

After 13 weeks of oral administration of control, ARA, or DHA oils, we measured the effects of the cytotoxic activity of NK cells from the spleen on YAC-1 cells. The NK cell activity in the ARA group of lymphocytes *versus* YAC-1 cells (5:1) was significantly suppressed compared with that in the control group. Moreover, the NK cell activity in the DHA group was slightly increased, but not significantly compared with that in the control group (Figure 1).

### 3.2. Analysis of Fatty Acid Metabolites

**Figure 2:**
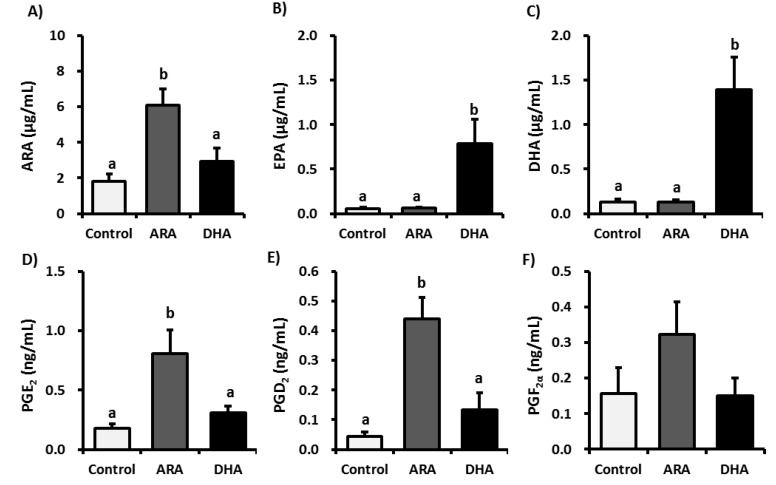
Plasma levels of free PUFAs and PGs in control, ARA, or DHA oil-treated rats. Plasma samples were subjected to LC-MS/MS lipidomic analysis. The graph presents the concentrations of: (**A**) ARA, (**B**) EPA, (**C**) DHA, (**D**) PGE_2_, (**E**) PGD_2_, and (**F**) PGF_2α_ in the rat plasma. The values are expressed as the mean ± S.E. (*n* = 8). Samples were analyzed using a one-way ANOVA followed by a Bonferroni *post hoc* test. a, b Different letters indicate statistical differences at *p* < 0.05.

**Figure 3:**
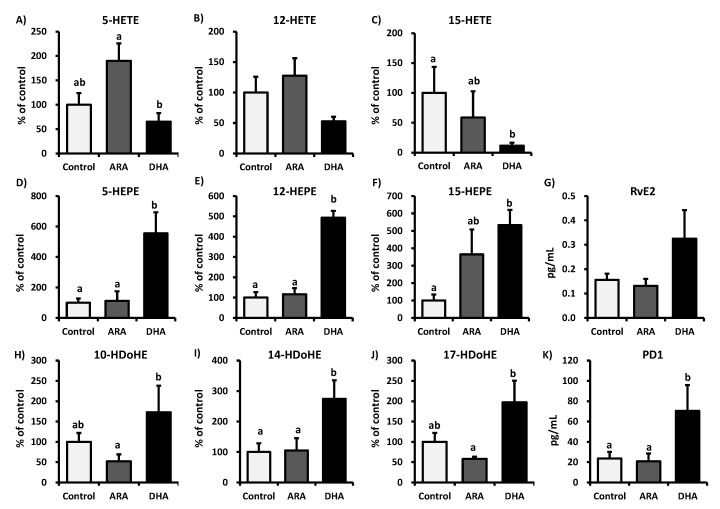
Plasma levels of ARA, EPA, and DHA metabolites generated by LOXs. Plasma samples were subjected to LC-MS/MS lipidomic analysis. The values are expressed as the mean ± S.E. (*n* = 8) percentages relative to the control. The graph presents the plasma levels of the metabolites from ARA, EPA, and DHA: (**A**) 5-HETE, (**B**) 12-HETE, (**C**) 15-HETE, (**D**) 5-HEPE, (**E**) 12-HEPE, (**F**) 15-HEPE, (**G**) RvE2, (**H**) 10-HDoHE, (**I**) 14-HDoHE, (**J**) 17-HDoHE, and **K**) PD1. Samples were analyzed using a one-way ANOVA followed by a Bonferroni *post hoc* test. a, b Different letters indicate statistical differences at *p* < 0.05.

Figure 2 lists the free form ARA, EPA, DHA, and ARA-derived eicosanoids detected in the plasma. The plasma levels of ARA, PGE_2_, and PGD_2_ were significantly higher in the ARA group than in the control and DHA groups. However, DHA administration had no significant effect on the levels of ARA-derived eicosanoids. In contrast, the free form EPA and DHA levels in the plasma were significantly higher in the DHA group than in the control and ARA groups. Figure 3 presents the plasma levels of the metabolites produced from ARA, EPA, and DHA by LOXs. ARA administration tended to increase the plasma levels of 5-HETE but not 12-HETE and 15-HETE, whereas there were no significant effects on the EPA- and DHA-derived metabolite levels. However, DHA administration significantly decreased the plasma levels of 15-HETE but not 5-HETE and 12-HETE. In addition, DHA administration significantly increased the plasma levels of 5-HEPE, 12-HEPE, 15-HEPE, 14-HDoHE, and PD1, whereas the plasma levels of RvE2, 10-HDoHE, and 17-HDoHE also tended to increase, although not significantly, compared with those in the control group. These results suggest that the administration of ARA or DHA does not induce LOXs.

### 3.3. Analysis of Inflammatory Cytokines in Plasma

After 13 weeks of oral administration of ARA or DHA, we measured the plasma levels of the inflammatory cytokines. IL-1β, IL-4, IL-6, IL-10, IL-13, and TNF-α were not affected by the administration of ARA and DHA in young rats (Table 3).

**Table 3:** Inflammatory cytokine levels in plasma.

(ng/mL)	Control	ARA	DHA
IL-1β	107.4 ± 6.08	146.9 ± 31.1	91.26 ± 8.42
IL-6	42.07 ± 11.6	27.87 ± 9.47	26.69 ± 8.74
TNF-α	20.84 ± 2.96	20.99 ± 2.17	21.65 ± 2.23
IL-4	6.99 ± 0.60	6.99 ± 0.62	6.65 ± 0.79
IL-10	129.4 ± 16.9	112.2 ± 8.25	112.8 ± 16.6
IL-13	14.63 ± 0.80	17.54 ± 1.25	13.20 ± 2.02

Note: IL, interleukin; TNF, tumor necrosis factor. Values are the means ± S.E. (*n* = 8).

## 4. Discussion

NK cell activity is important for suppressing cancer through the innate immune system. NK cells are activated by macrophages and various pro-inflammatory cytokines such as IL-2, IL-12, and TNF-α. NK cell activity is associated with innate immune reactions and inflammatory reactions [28,29]. In this study, we investigated the effects of long-term administration of ARA and DHA on the NK cell activity levels and inflammatory process in young rats. Long-term ARA administration in young rats suppressed the activity of NK cells from spleen lymphocytes, suggesting that ARA has the potential to inhibit the cancer cell cytotoxic activity of NK cells and reduce the resistance to cancer.

ARA is converted to pro-inflammatory PGs, which induce inflammation. In the present study, long-term ARA administration had no effect on pro-inflammatory cytokines in the plasma. In contrast, ARA administration significantly increased the plasma levels of ARA, PGD_2_, and PGE_2_, which suggests that ARA itself might not induce inflammation. PGE_2_ has an initial pro-inﬂammatory effect, which is followed by a role in the resolution of inﬂammation by the inhibition of 5-LOX and the induction of lipoxin production [30,31,32,33]. PGE_2_ mediates active inflammation, promotes local vasodilatation and local attraction, and activates neutrophils, macrophages, and mast cells in the early stages of inflammation. In contrast, PGE_2_ can promote the induction of suppressive IL-10 and directly suppresses the production of multiple pro-inflammatory cytokines, which allow it to limit nonspecific inflammation, thereby promoting the immune suppression associated with chronic inflammation and cancer. Recent studies have revealed that PGE_2_ down-regulates IL-2-activated LAK cell cytotoxicity via the PGE_2_ receptor, EP2. PGE_2_ inhibits lymphocyte proliferation, NK cell activity, and the production of Th1 cytokines (IL-2, IFN-γ) [34,35,36]. PGE_2_ also reduced the amount of IFN-γ and TNF-α secretion by NK cells, in human NK cells, IFN-γ secretion was suppressed by PGE_2_ in a dose-dependent manner [24]. In addition, PGE_2_ inhibits MHC II expression and the production of TNF-α and IL-1β by monocytes and macrophages. The presence of PGE_2_ inhibits the NK cell-mediated cytotoxicity against YAC-1 tumor targets [37,38]. Moreover, PGD_2_ regulates the immune function via D prostanoid receptors and chemoattractant receptor-like molecules on Th2 cells, which inhibit IL-12 production by dendritic cells and INF-γ production by T cells. It was reported that PGD_2_ inhibits the cytotoxic function of human NK cells directly via D prostanoid receptors [39,40,41,42,43]. In the present study, long-term ARA administration inhibited NK cell activity and increased the plasma levels of PGE_2_ and PGD_2_; this suggests that the immune response might be suppressed during chronic inflammation or cancer to maintain high levels of PGE_2_ and PGD_2_ in the plasma. The anti-inflammatory properties of EPA and its metabolites are beneficial during chronic inflammation, but they could suppress the inflammatory response required to attack acute viral infections [44]. EPA but not DHA has been reported to decrease the NK cell activity in rat lymphocytes, a mixture of NK cells and macrophages, and in human peripheral blood mononuclear cells, which are a mixture of lymphocytes such as NK cells and monocytes [45,46,47,48,49]. In this study, long-term DHA administration increased the plasma free EPA levels slightly but not significantly (Figure 2), which in turn increased the NK cell activity from spleen lymphocytes, particularly in cases with a low lymphocyte *versus* YAC-1 ratio. The differences between these groups might be because of the low EPA dose administered in our study.

PUFAs are present in dietary supplements for babies and adults. ARA is an essential fatty acid, which is stored in the cell membrane, and it is present in high levels in the brain and other tissues. Supplementation with ARA is considered to have beneficial effects on cognitive function and communication ability. However, long-term ARA administration has been associated with cancer development or the stimulation of inflammatory conditions [17,18,19]. In a previous study, ARA supplementation for four weeks in elderly Japanese subjects did not increase the levels of ARA metabolites, which suggests that it does not induce inflammation [50]. In the present study, we demonstrated that long-term ARA administration did not induce inflammation, but our results suggested that it might disrupt the immune system, thereby making it more vulnerable to infection or cancer. DHA and EPA, which are present in fish oil, are also essential fatty acids and they can suppress the serum triglyceride levels as well as improve cognitive function. In contrast to ARA, long-term DHA administration did not affect the plasma levels of free ARA, PGE_2_, PGD_2_, and PGF_2α_. Moreover, DHA had no effect on the plasma levels of inflammatory cytokines, and it tended to increase NK cell activity, thereby indicating that DHA does not disrupt the immune system. There were some limitations in the present study because we used normal young rats to test the effects of ARA or DHA administration. It is necessary to test an inflammatory model or cancer model animals to confirm the effects of long-term administration of ARA or DHA on the immune system, particularly NK cell activity in the spleen.

## 5. Conclusions

In conclusion, long-term ARA administration tended to decrease the innate immunity to cancer, although the inflammatory cytokine levels were unaltered in the plasma. The higher plasma levels of PGE_2_ and PGD_2_ might explain the decreased NK cell activity in the spleen. In future, we plan to elucidate the mechanism by which long-term ARA therapy controls the NK activity. Furthermore, we also plan to test a dosage range of ARA and a supplementary time period.
